# Curcumin Generates Oxidative Stress and Induces Apoptosis in Adult *Schistosoma mansoni* Worms

**DOI:** 10.1371/journal.pone.0167135

**Published:** 2016-11-22

**Authors:** Daniela de Paula Aguiar, Mayara Brunetto Moreira Moscardini, Enyara Rezende Morais, Renato Graciano de Paula, Pedro Manuel Ferreira, Ana Afonso, Silvana Belo, Amanda Tomie Ouchida, Carlos Curti, Wilson Roberto Cunha, Vanderlei Rodrigues, Lizandra Guidi Magalhães

**Affiliations:** 1 Núcleo de Pesquisa em Ciências Exatas e Tecnológicas, Universidade de Franca, Franca, Brazil; 2 Instituto de Genética e Bioquímica, Universidade Federal de Uberlândia, Patos de Minas, Brazil; 3 Departamento de Bioquímica e Imunologia, Universidade de São Paulo, Ribeirão Preto, Brazil; 4 Global Health and Tropical Medicine, GHTM, UEI Medical Parasitology, Instituto de Higiene e Medicina Tropical, IHMT, Universidade Nova de Lisboa, UNL, Lisbon, Portugal; 5 Instituto de Química de São Carlos, Universidade de São Paulo, São Carlos, Brazil; 6 Departamento de Morfologia e Patologia, Universidade Federal de São Carlos, São Paulo, Brazil; 7 Departamento de Física e Química, Faculdade de Ciências Farmacêuticas de Ribeirão Preto, Universidade de São Paulo, Ribeirão Preto, São Paulo, Brazil; Alexandria University, EGYPT

## Abstract

Inducing apoptosis is an interesting therapeutic approach to develop drugs that act against helminthic parasites. Researchers have investigated how curcumin (CUR), a biologically active compound extracted from rhizomes of *Curcuma longa*, affects *Schistosoma mansoni* and several cancer cell lines. This study evaluates how CUR influences the induction of apoptosis and oxidative stress in couples of adult *S*. *mansoni* worms. CUR decreased the viability of adult worms and killed them. The tegument of the parasite suffered morphological changes, the mitochondria underwent alterations, and chromatin condensed. Different apoptotic parameters were determined in an attempt to understand how CUR affected adult *S*. *mansoni* worms. CUR induced DNA damage and fragmentation and increased the expression of *SmCASP3/7* transcripts and the activity of Caspase 3 in female and male worms. However, CUR did not intensify the activity of Caspase 8 in female or male worms. Evaluation of the superoxide anion and different antioxidant enzymes helped to explore the mechanism of parasite death further. The level of superoxide anion and the activity of Superoxide Dismutase (SOD) increased, whereas the activity of Glutathione-S-Transferase (GST), Glutathione reductase (GR), and Glutathione peroxidase (GPX) decreased, which culminated in the oxidation of proteins in adult female and male worms incubated with CUR. In conclusion, CUR generated oxidative stress followed by apoptotic-like-events in both adult female and male *S*. *mansoni* worms, ultimately killing them.

## Introduction

Schistosomiasis is a neglected tropical disease that affects more than 250 million people worldwide. It causes over 300,000 deaths annually and leads to loss of 1.53 million active lives in 74 endemic countries per year due to disability of adjusted life (DALYs) [1]. Schistosome parasites of the dioecious trematode flatworm type cause this disease. These parasites have a very complex life cycle. *Schistosoma mansoni* is one of the etiological agents of human schistosomiasis, which is currently endemic in Africa, in the Middle East, in the Caribbean, and in South America [1].

There is no effective vaccine against schistosomiasis, and treatment is currently limited to Praziquantel (PZQ). PZQ is effective against all *Schistosoma* species: it is safe, mostly available, and inexpensive, and it dismisses the need for direct medical supervision [2,3]. However, reduced cure rates and treatment failures have been reported. PZQ also presents low efficacy against juvenile worms (aged between 7 and 28 days), and multiple failures in preventing reinfection have been reported [4–6]. The fact that treatment of schistosomiasis is limited to one single drug has made the World Health Organization (WHO) urge researchers to find an alternative to PZQ [1,7].

Inducing apoptosis is an interesting therapeutic strategy to develop drugs. This strategy still has to be explored in the treatment of diseases caused by metazoan helminthic parasites [8]. Apoptosis is the major form of programmed cell death in metazoan organisms, and it plays a critical role in normal development, tissue homeostasis, and immunity. Impaired regulation of apoptosis contributes to various pathological states [9]. There are two major apoptosis pathways: an extrinsic pathway (or death receptor) and an intrinsic (or mitochondrial) pathway. In vertebrates, engagement of ‘death receptors’ of the family of tumor necrosis factor receptors (TNFR) present on the surface of the cell membrane triggers the extrinsic pathway [9]. Many intracellular signals (developmental differences, cytotoxic insults, or several cellular stresses) that act upon the family of BCL-2 proteins activate the intrinsic pathway and then control the integrity of the mitochondrial outer membrane through various complex interactions [10,11]. The triggered apoptotic pathways converge upon activation of effector caspases, which underlie the morphological features of apoptotic cells. The so-called “death receptors” (TNFR family) have not yet been described in the transcriptome or in the genome of *S*. *mansoni* [12–14]. However, the presence of the TNFR2 receptor (with non-death-domain) and of other genes such as the Fas death domain-associated protein (FADD) has already been described in this parasite [12–15]. The Bcl-2 family has been identified and characterized, which has provided molecular evidence of an intrinsic apoptosis pathway in parasitic flatworms [16,17].

Numerous plants have been chemically and biologically investigated to discover useful herbal preparations or natural active constituents that might be used as lead compounds to develop new drugs. Such new drugs could be potentially applied in the treatment of neglected tropical diseases (NTD), including schistosomiasis [18,19]. Curcumin (1,7-bis(4-hydroxy-3-methoxyphenyl)-1,6 heptadiene-3,5-dione) (CUR) is the major curcuminoid compound extracted from *Curcuma longa* L., a plant that possesses many pharmacological and biological activities [20], including antiparasitic action [21–25]. Previous studies by our group have shown that CUR can act against adult *S*. *mansoni* worms *in vitro* [21,26], and that it can regulate the expression of 2,374 genes, including the genes of caspase 8 (*SmCASP8*) [27]. Other studies associated with CUR have induced the generation of reactive oxygen species (ROS) in several cancer cell lines, which also leads to cell apoptosis by the intrinsic pathway [28–30]. Additionally, studies have shown that CUR can generate ROS in the nematodes *Setaria cervi* and *S*. *digitata*, and that it participates in the induction of apoptosis [8,31].

In this paper, we have investigated how CUR affects the induction of apoptosis and oxidative stress in pairs of adult *S*. *mansoni* worms. To this end, we have analyzed the viability, the alterations in tegument and organelles, the DNA fragmentation and damage, and the expression and activity of caspases of couples of adult worms treated with CUR. We have also evaluated different parameters of oxidative stress including production of the superoxide anion, the activities of various enzymatic antioxidants, and the levels of protein carbonyls.

## Materials and Methods

### Ethics statement

Six-week-old female BALB/c mice weighing 20–25 g were obtained from the Animal House of the University of São Paulo, Brazil. All the animals were acclimated for one week before the experiments began. The mice were housed in plastic bins with wire tops and wood chip bedding (five mice per bin) at the animal research facility of the university. They were placed under controlled conditions of temperature (22±2°C) and humidity (50±10%) and a 12-h light–dark cycle. They were fed standard rat chow (Labina, São Paulo, Brazil) with access to water *ad libitum*. The Ethics Committee for Animal Care of the University of Franca authorized all the experiments (Approval number: 028/12). All the animals were handled by using good animal practice as defined by the University of Franca in agreement with the Brazilian legislation (CEUA, 11.794/2008).

### Drugs

Curcumin (1,7-bis(4-Hydroxy-3-methoxyphenyl)-1,6-heptadiene-3,5-dione) (CUR) and Praziquantel (2-(Cyclohexylcarbonyl)-1,2,3,6,7-11b-hexahydro-4H-pyrazino[2,1-a]isoquinolin-4-one) (PZQ) were purchased from Sigma-Aldrich, St Louis, USA. Stock sterile solutions of CUR and PZQ at 100 mM were then prepared in 10% dimethyl sulfoxide (DMSO) (Sigma-Aldrich).

### Parasite maintenance, recovery, and culture

The LE (Luiz Evangelista) strain of *S*. *mansoni* was used in all the experiments. The life cycle of the parasite was routinely maintained by passage through *Biomphalaria glabrata* snails and BALB/c mice at the animal house of the University of Franca. Cercariae were obtained from infected snails exposed to light for 1 h after 38–45 days of infection according to the standard procedures at our laboratory. Each mouse was percutaneously infected with 200±10 cercariae. After 50±2 days of infection, the mice were euthanized, and the couples of adult *S*. *mansoni* worms were recovered under aseptic conditions by perfusion of their livers and mesenteric veins [32]. The worms were washed in RPMI 1640 medium (Inlab Diagnonóstica, São Paulo, BRA) supplemented with penicillin (100 UI/mL), streptomycin (100 μg/mL), and 10% bovine fetal serum (Cultilab, Campinas, BRA) prior to use. Before the experiments, one or ten couples of adult *S*. *mansoni* worms were placed in every well of a 24-well plate or in a 25-cm^2^ culture flask containing 2 mL or 20 mL of the same culture medium, respectively, and incubated at 37°C in humid atmosphere containing 5% CO_2_ for 24 h, for adaptation.

### Assay for parasite viability

The MTT (3-(4,5-Dimethylthiazol-2-yl)-2,5-Diphenyltetrazolium Bromide) colorimetric assay was used to determine the viability of the parasite by the method described by Comley et al. (1989) [33]. One couple of adult *S*. *mansoni* worms was placed in each well of a 24-well culture. Then, CUR was added to a final concentration of 1.56 to 100 μM and incubated for 6, 12, or 24 h. After incubation, female and male *S*. *mansoni* worms (separated either by action of CUR or manually, after treatment) were individually placed into wells (96-well plates) containing 5 mg of MTT/mL in phosphate buffered saline (PBS) (Sigma-Aldrich) at 37°C for 2 h. The solution was carefully removed and replaced with DMSO, and the worms were allowed to stand in DMSO at room temperature for 1 h. The absorbance was read at 550 nm with a spectrophotometer (Biochrom Corp, Miami, USA). The experiment was repeated three times, and ten couples of adult worms were evaluated in each experiment. For the negative control group, couples of adult worms were incubated with RMPI 1640 medium or with RPMI 1640 medium containing 0.1% DMSO. For the positive control group, couples of adult worms were incubated with PZQ (1.56 μM) or heat-killed at 56°C.

An additional criterion for viability was supported by microscopic observation of *S*. *mansoni* adult worms that focused on changes in the motility of worms and on the occurrence of death based on standard procedures for the screening of compounds of the WHO-TDR [34]. Couples of adult worms were incubated for 6, 12, or 24 h in the same conditions described above and monitored with an inverted microscope (Carl Zeiss, Göttingen, DEU). The phenotypic changes were scored on the basis of a viability scale of 0 to 3: (3 = total activity, 2 = slow activity, 1 = minimal activity, 0 = worm death—death was defined as the absence of movement for at least 2 min of examination). After the last observation period (24 h), the culture medium was removed, fresh culture medium without CUR was added, and motility was re-examined for up to 24 h. Additionally, the separation of couples of adult worms was assessed. The experiment was repeated three times, and ten couples of adult worms were evaluated in each experiment. For the negative control group, couples of adult worms were incubated with RMPI 1640 medium or in RPMI 1640 medium with 0.1% DMSO. For the positive control group, couples of adult worms were incubated with PZQ (1.56 μM). Lethal Concentration (LC_50_) values were calculated from a nonlinear regression dose–response inhibition graph.

### Transmission Electron Microscopy (TEM)

To verify the ultrastructural alterations caused by CUR, couples of adult worms were placed in 25-cm^2^ culture flasks (ten couples of adult worms were placed in each culture flask) as previously described. Then, CUR was added to a final concentration of 50 μM (next to the LC_50_ value for the female and male worms at 24 h), and the cultures were incubated for 6, 12, or 24 h. After incubation, female and male *S*. *mansoni* worms (separated either by action of CUR or manually, after treatment) were washed three times with phosphate buffer and fixed in 2.5% glutaraldehyde-phosphate buffer (0.2 M, pH 7.4) at room temperature for 2 h. The worms were post-fixed with 1% osmium tetroxide (Sigma-Aldrich) in the same buffer at 4°C, for 2 h. The worms were dehydrated in graded ethanol and embedded in Araldite 6005 resin (EMS). Ultrathin sections of the schistosomes were stained with 0.5% uranyl acetate (Sigma-Aldrich) and 0.3% lead citrate (Sigma-Aldrich). Ultrastructural features of the schistosome sections were examined with a TEM microscope (JEOL Model JEM-100CXII equipped with a Hamamatsu ORCA-HR digital camera, Tokyo, JPN). The experiment was repeated twice, and ten couples of adult worms were evaluated in each experiment. For the negative control group, couples of adult worms were incubated with RMPI 1640 medium with 0.1% DMSO.

### Detection of DNA fragmentation

Couples of adult worms were placed in 25-cm^2^ culture flasks (ten couples of adult worms were placed in each culture flask) as previously described. Then, CUR was added to a final concentration of 25 or 50 μM, and the cultures were incubated for 24 h. After incubation, the genomic DNA from female and male *S*. *mansoni* worms (separated either by action of CUR or manually, after treatment) was extracted as described by Sambrook, Russel (2001) [35]. The concentration of DNA was measured on a spectrophotometer (NanoVue Plus Spectrophotometer, GE Healthcare, Buckinghamshire, ING), and 600 ng of DNA was analyzed by electrophoresis in 2% agarose gel containing 1% GelRed (1:500) (Biotium, Hayward, EUA) and subsequently analyzed with a SmartView Pro Imager System (Major Science, California, EUA). The experiment was repeated twice. Ten pairs of adult worms were evaluated in each experiment. For the negative control group, pairs of adult worms were incubated with RMPI 1640 medium with 0.1% DMSO.

### Terminal deoxynucleotidyl transferase-mediated dUTP-biotin nick end labeling staining of apoptotic nuclei (TUNEL)

Breaks in DNA strands were detected in CUR-treated adult worms by using the Terminal Deoxynucleotidyl Transferase dUTP Nick End Labelling (TUNEL) method and the DeadEnd Colorimetric TUNEL System (Promega, Madison, USA). Briefly, pairs of adult worms were placed in 25-cm^2^ culture flasks (ten couples of adult worms were placed in each culture flask), as previously described. Next, CUR was added to a final concentration of 50 μM, and the culture was incubated for 24 h. After incubation, female and male *S*. *mansoni* worms (separated either by action of CUR or manually, after treatment) were fixed in 4% paraformaldehyde at 4°C for 12 h, embedded in paraffin, and cut into 5 μm-thick sections. Paraffin was removed from parasite sections in xylene, and the sections were rehydrated in graded ethanol and distilled water. After being rinsed three times with phosphate buffered saline (PBS), the slides were permeabilized with proteinase K (20 mg/mL) at room temperature for 15 min. The slides were incubated with equilibrium buffer, and the fragmented DNA was labeled with a biotinylated nucleotide mix in the presence of recombinant deoxynucleotidyl transferase for 1 h in a humidified chamber. Then, cell apoptosis was assessed with a terminal deoxynucleotidyl transferase dUTP nick end labeling kit according to the manufacturer’s instructions. Microscopy was used (Carl Zeiss). In histological sections, the apoptotic index, defined as the percentage of apoptotic cells, was used as a quantitative measure of apoptosis. The apoptotic index was determined as follows: (number of TUNEL-positive cells/total number of cells) x 100. The experiment was repeated three times, and ten couples of adult worms were evaluated in each experiment. For the negative control group, couples of adult worms were incubated with RMPI 1640 medium with 0.1% DMSO.

### Comet assay

The comet assay was performed according to Azqueta et al. (2014) [36]. Couples of adult worms were placed in 25-cm^2^ culture flasks (ten couples of adult worms were placed in each culture flask) as previously described. Then, CUR was added to a final concentration of 25 or 50 μM, and the cultures were incubated for 24 h. After incubation, female and male *S*. *mansoni* worms (separated either by action of CUR or manually, after treatment) were sliced with a scalpel in Trizol reagent (Invitrogen, Carlsbad, EUA) and then agitated by vortex for 15 min. The cell suspension was centrifuged at 200*g* and 4°C for 10 min. Next, single cells were embedded in a 1% low-melting agarose in water and transferred to microscope slides pre-coated with 1% normal agarose. After brief solidification of agarose at 4°C, the coverslip was removed, and the slides were immersed in lysis solution (2.5 M NaCl, 0.1 M Na_2_−EDTA, 10 mM Tris, pH 10 and 10% DMSO and 1% Triton X-100) for 16 h, to promote cell lysis and to allow DNA to unfold. The slides were then removed from the lysis solution and placed on a horizontal gel electrophoresis unit containing alkaline buffer (1mM Na_2_−EDTA and 300 mM NaOH). The slides were allowed to set in this buffer for 20 min, to unwind the DNA before electrophoresis. Electrophoresis was conducted at 25 V for the next 20 min. After that, the slides were washed with 0.4 M Tris (pH 7.5), to remove the alkali and detergents. The slides were then stained with 20 μg/ml ethidium bromide in a solution of distilled water, and each slide was visualized under a fluorescent microscope (Carl Zeiss). For each slide, 50 random cells were analyzed visually, and each comet class had a value lying between 0 and 4: (0) undamaged cells (all the DNA was located in the head) and (4) maximum damage (almost all the DNA was located in the tail). The total score was calculated by multiplying the percentage of damaged nucleoids by the value of the respective comet class (0, 1, 2, 3, or 4). A value of 0 indicated no damage, and a value of 400 corresponded to maximum damage. The experiment was repeated three times, and ten couples of adult worms were evaluated in each experiment. For the negative control group, couples of adult worms were incubated with RMPI 1640 medium with 0.1% DMSO.

### Preparation of RNA and analysis of RNA expression by quantitative RT-PCR

Couples of adult worms were placed in 25-cm^2^ culture flasks (ten couples of adult worms were placed in each culture flask) as previously described, and they were incubated with CUR at 25 or 50 μM for 24 h. After incubation, the total RNAs from female and male *S*. *mansoni* worms (separated either by action of CUR or manually, after treatment) were isolated by using a combination of the reagent Trizol (Invitrogen, Carlsbad, EUA) for extraction and the PureLink^TM^Micro-to-Midi Total RNA Purification System (Invitrogen) for purification. For cDNA synthesis, 1 μg of total RNA was treated with 4 U of DNase I (Promega) and used as a template to synthesize cDNA with an oligodT primer from the ThermoScript^TM^ RT-PCR System (Invitrogen). The manufacturer's protocol was followed. The specific primers for *SmCASP 3*, *7*, and *8* used in this study had been previously described by Dubois et al. (2009) [37] and Morais et al. (2013) [27] as follows: *SmCASP3* forward, 5´-TTTGCGGTCAATGAAGAAATAAAC-3´, reverse 5´-AAGAGCGAAACACAATCGTGC-3´; *SmCASP7* forward, 5´-CGTGACCATGATTGTTTCGC-3´, reverse 5´-GCAATGATACGATCCACGGG-3´; *SmCASP8* forward, 5´- GCGATGAATTCTAAGGGGAAG-3´, reverse 5´- GCACAATGTAGTGCCGTATTTC-3´. Specific primers for *S*. *mansoni SmGAPDH* were used as endogenous control (forward, 5′-TCGTTGAGTCTACTGGAGTCTTTACG-3′ and reverse 5′ AATATGAGCCTGAGCTTTATCAATGG-3′). In previous studies, these primers had been used to verify the expression of the transcripts in adult *S*. *mansoni* worms incubated with CUR [38, 39]. To confirm the specificities of the primers, the PCR products were sequenced in the ABI 3100 automated sequencer (Applied Biosystems) by using a Dye Terminator kit.

The reactions were performed in triplicate and carried out by using the 7500 real-time PCR system (Applied Biosystems, Foster City, EUA). The PCR efficiency (E) was determined for both primer sets by plotting cycle thresholds from a tenfold serial dilution of cDNA and by introducing the slope in the equation E = 10^(–1/slope)^. For PCR amplification, the samples were incubated at 95°C for 10 min and submitted to 40 cycles of 95°C for 15 s and 60°C for 1 min. To evaluate gene expression, the total reaction volume was 10 μL containing each primer at 100 nM, 5 μl of SYBR green PCR (Aplied Biosystems), and 1 μl containing 200 ng of cDNA as template (or water as negative control). The gene expression was calculated by the comparative Ct method (2^-ΔΔCT^ method) [40], and the data were normalized relative to an endogenous standard gene (*SmGPDH*) and then calculated as the fold change in the levels of expression relative to the control group (adult worms in RPMI 1640 medium with 0.1% DMSO).

### Activities of Caspase 3 and 8

Couples of adult worms were placed in 25-cm^2^ culture flasks (ten couples of adult worms were placed in each culture flask), as previously described. Then, CUR was added to a final concentration of 25 or 50 μM, and the cultures were incubated for 24 h. After incubation, female and male *S*. *mansoni* worms (separated either by the action of CUR or manually, after treatment) were homogenized in an extraction buffer (5 mM EDTA, 150 mM NaCl, 20 mM Tris 7.5, 1 mM DTT, 1% Triton-X100, and 50 μM cathepsin inhibitor K77111) [41, 42] by using a sonicator (four two-minute cycles with pulses of 0.75 s and 40% amplitude), which was followed by centrifugation at 5000 g and then at 15,500 *g* at 4°C for 15 min. Finally, the supernatant was collected, and the protein content was determined by using the Protein Assay Reagent Coomassie Plus (Thermo Scientific, Waltham, EUA) according to the manufacturer’s instructions.

The activity of Caspase 3 was measured by using the Acetyl-Asp-Glu-Val-Asp-p-nitroaniline (p-Na) substrate (Sigma- Aldrich) according to the manufacturer’s instructions. Samples (50 μg of crude extracts) and 2 mM substrate were added to 200 μL of a reaction buffer [120 mM HEPES (pH 7.4), 0.1% CHAPS, 5 mM DTT, and 2 mM EDTA] and incubated at 37°C for 90 min. The absorbance was read at 405 nm with the aid of a spectrophotometer (Biochrom). The activity of Caspase 8 was measured with the Caspase 8 Assay Kit (Sigma-Aldrich) according to the manufacturer’s instructions. Samples (50 μg of crude extracts) and 150 μM caspase fluorigenic substrate (Ac-IETD-AMC) were added to 100 μL of reaction buffer and incubated at 25°C for 1 h. The fluorescence was determined by using a Sinergy 2 multi-mode microplate reader (BioTek, USA). The AMC release was determined at an excitation wavelength of 360 nm and at an emission wavelength of 440 nm. The experiments were repeated three times, in triplicate. For the negative control group, couples of adult worms were incubated with RPMI 1640 medium with 0.1% DMSO. The reaction buffer was used for the blank control.

### Determination of the level of superoxide anion

The level of superoxide anion was measured by using the colorimetric Nitroblue Tetrazolium (NBT) assay as described previously by Choi et al. (2006) [43]. Briefly, adult worm pairs were placed in culture plates (one couple of adult worm was placed in each well) as previously described and incubated with CUR at concentrations ranging from 12.5 to 100 μM for 6, 12, or 24 h. After incubation, female and male *S*. *mansoni* worms (separated either by action of CUR or manually, after treatment) were individually placed in wells (96-well plates) containing 2% NBT solution (Sigma-Aldrich) at room temperature for 1 h. After that, the adult worms were washed twice with PBS and once with methanol. The resulting formazan was solubilized by addition of 140 μl of 2 M KOH and 140 μl of DMSO with gentle shaking at room temperature for 10 min. The absorbance was read on a microplate reader at 620 nm (Biochrom). The experiments were repeated three times, and ten couples of adult worms were evaluated in each experiment. For the negative control group, couples of adult worms were incubated with RMPI 1640 with 0.1% DMSO. For the positive control group, couples of adult worms were incubated with RPMI 1640 medium with 100 μM hydrogen peroxide,

### Determination of the activities of the antioxidant enzymes

#### Preparation of the crude extract of adult worms

Briefly, couples of adult worms were placed in 25-cm^2^ culture flasks (ten couples of adult worms were placed in each culture flask), as previously described, and were incubated with CUR at 25 or 50 μM, in RPMI 1640 medium plus 0.1% DMSO (negative control) or in RPMI 1640 medium with 100 μM hydrogen peroxide (positive control) for 6, 12, or 24 h. After this period, female and male *S*. *mansoni* worms (separated either by action of CUR or manually, after treatment) were sonicated (four two-minute cycles with pulses of 0.75 s and 40% amplitude) in a phosphate buffer (pH 7.4) at 4°C. Homogenates were centrifuged at 5000 *g* followed by centrifugation at 15500 *g* at 4°C for 15 min, and the concentration of protein was determined with a Pierce BCA® Protein Assay Kit (Thermo Scientific) according to the manufacturer’s instructions. The clear supernatant was stored at –70°C until use. The crude extracts were prepared in triplicate for each group.

#### Activity of Superoxide Dismutase (SOD)

The activity of SOD was measured with the SOD determination Kit (Sigma-Aldrich) and 50 μg of the crude extract. The assay was performed in a 96-well plate according to the manufacturer's instruction, and the absorbance at 450 nm was read with a spectrophotometer (Biochrom). A standard linear regression curve of SOD was prepared according to the protocol and was used to detect the activity of SOD. The experiments were performed three times, in triplicate. The blank control consisted of reaction buffer without crude extract.

#### Activity of Glutathione-S-transferase (GST)

The activity of GST was determined according to the method described by Habig et al. (1974) [44]. The assay was performed in a 96-well plate containing 50 μg of crude extracts and reaction solution [50 mM 1-chloro-2,4-ditnitrobenzene (CDNB) and 5 mM glutathione (GSH) in 0.1 M phosphate buffer]. The reaction was incubated at 25°C for 5 min, and the absorbance was read at 340 nm with a spectrophotometer (Biochrom) for 5 min. The experiments were performed three times, in triplicate. The blank control consisted of reaction solution without crude extract. One unit of enzyme activity was defined as the amount of enzyme that catalyzed the oxidation of 1 mmol of substrate (CDNB)/min at 25°C.

#### Activity of Glutathione Reductase (GR)

The activity of GR was assayed according to the method described by Carlberg, Mannervik, 1981 [45]. The reaction assay was performed in a 96-well plate and was initiated by addition of 0.1 mM NADPH to a mixture of crude extract (50 μg) in 50 mM potassium phosphate buffer pH 7.4 containing 2 mM EDTA and 0.5 mM GSSG. The absorbance was read at 340 nm with a spectrophotometer (Biochrom) for 3 min. The experiments were performed three times, in triplicate. The blank control consisted of reaction buffer without crude extract. One unit of GR activity was defined as the amount of enzyme that catalyzed the reduction of 1 mmol of NADPH per minute.

#### Activity of Glutathione Peroxidase (GPX)

Mei et al. (1996) assessed the activity of GPX [46] in a coupled reaction that detected changes in the level of NADPH. The reaction was performed in a 96-well plate containing 50 μg of crude extracts, 0.5 mM cumene hydroperoxide (Sigma-Aldrich), 1 mM GSH, 0.1 U of GR, 5 mM K_2_HPO_4_, 0.2 mM EDTA, 0.2 mM NaN_3_, and 0.1 mM NADPH. The activity of the enzyme was determined from the linear portion of the absorbance read at 340 nm with a spectrophotometer (Biochrom). The experiments were performed three times, in triplicate. The blank consisted of reaction solution without crude extract. One unit of GPX activity was defined as the amount of enzyme required to oxidize 1 nmol of NADPH per min under the above-described assay conditions.

### Determination of the formation of protein carbonyl

The oxidation of protein was monitored by the content of protein carbonyl measured with 2,4–dinitrophenyl hydrazine (DNPH) [47]. Briefly, 10% cold trichloroacetic acid (TCA) was added to the extract at a 1:1 ratio, which was followed by centrifugation at 6000 *g* and 4°C for 5 min. Then, 10 mM DNPH dissolved in 2 N HCl was added to the pellet, which was allowed to stand in the dark at room temperature for 1 h with intermittent agitation by vortexing. The mixture was then centrifuged at 6000 *g* and 4°C for 5 min. The supernatant was discarded, and 20% TCA was added to the pellet, which was followed by centrifugation at 6000 *g* and 4°C for 5 min. The non-derivatized proteins in the pellet were washed 2–3 times with a 1:1 ethanol/ethyl acetate mixture until the pellet became clean. The final pellet was re-suspended in 6 M guanidium hydrochloride, and the absorbance at 370 nm was read with a spectrophotometer (Biochrom). The experiments were performed three times, in triplicate. The blank consisted of reaction solution without crude extract.

### Statistical analysis

The statistical tests were performed with the software Graphpad Prism (version 5.0). The data were statistically analyzed by one-way ANOVA analysis of variance and a *posteriori* Tukey’s test. For all the tests, a difference of *p* < 0.05 was considered significant. The LC_50_ (Lethal Concentration) value was calculated from nonlinear regression of dose–response inhibition graphs.

## Results

### CUR affects the viability of adult *S*. *mansoni* worms

**Fig 1:**
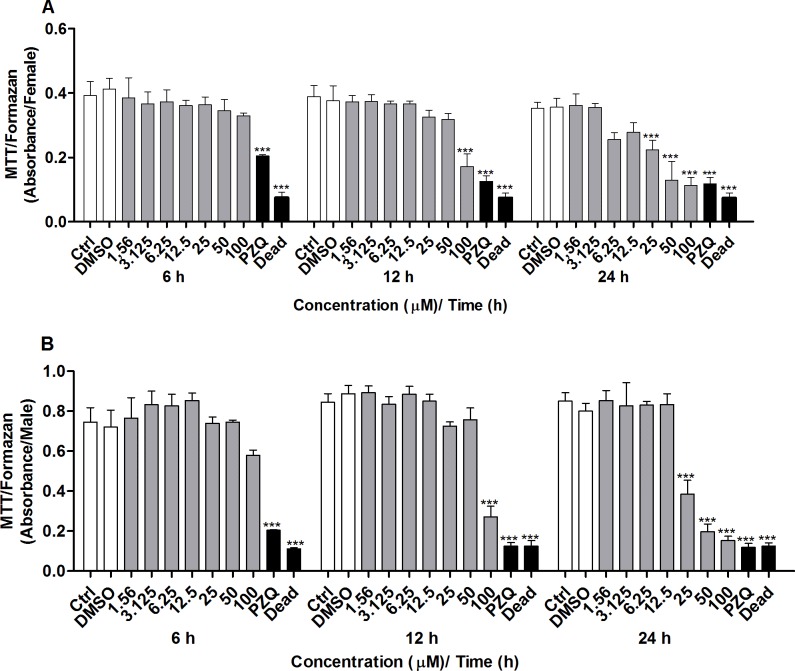
*In vitro* effect of CUR on the viability of adult *S*. *mansoni* worms as measured by MTT assay. Couples of adult worms were incubated with different concentrations of CUR for 6, 12, or 24 h. Adult (A) female and (B) male *S*. *mansoni* worms were separated, and the viability was measured by the MTT assay at 550 nm. In the negative control groups, couples of adult worms were incubated with RMPI 1640 medium or with RPMI 1640 medium with 0.1% DMSO. In the positive control groups, couples of adult worms were incubated with PZQ (1.56 μM) or heat-killed at 56°C. Values are expressed as the mean ± S.E.M of three independent experiments. An asterisk indicates statistically significant differences as compared to the negative control group (RPMI 1640 medium with 0.1% DMSO) (***p < 0.001).

Our group has already conducted an *in vitro* study into how CUR affects the viability of adult *S*. *mansoni* worm pairs (female and male placed in the same well) by using the MTT assay for 24 or 120 h [21]. Here, we have evaluated how CUR affects the viability of female and male *S*. *mansoni* worms separately. The worms were separated either by action of CUR at concentrations ranging from 1.56 to 100 μM or manually. The MTT assay [33] was conducted for 6, 12, or 24 h to assay the viability. Incubation of coupled adult worms with CUR for 6 h did not alter the viability of the female and male worms at any of the tested concentrations (Fig 1A and 1B). After 12 h of incubation, the viability of both female and male worms decreased considerably in the presence of CUR at 100 μM. After 24 h of incubation, the viability of both female and male worms diminished significantly in the presence of CUR at 25, 50, or 100 μM. In contrast, the couples of adult worms in the negative control groups (RPMI 1640 medium alone or in combination with 0.1% DMSO) exhibited normal viability, whereas the couples of adult worms in the positive control group (PZQ at 1.56 μM or heat-killed) were not viable (100% death) (Fig 1A and 1B).

**Fig 2:**
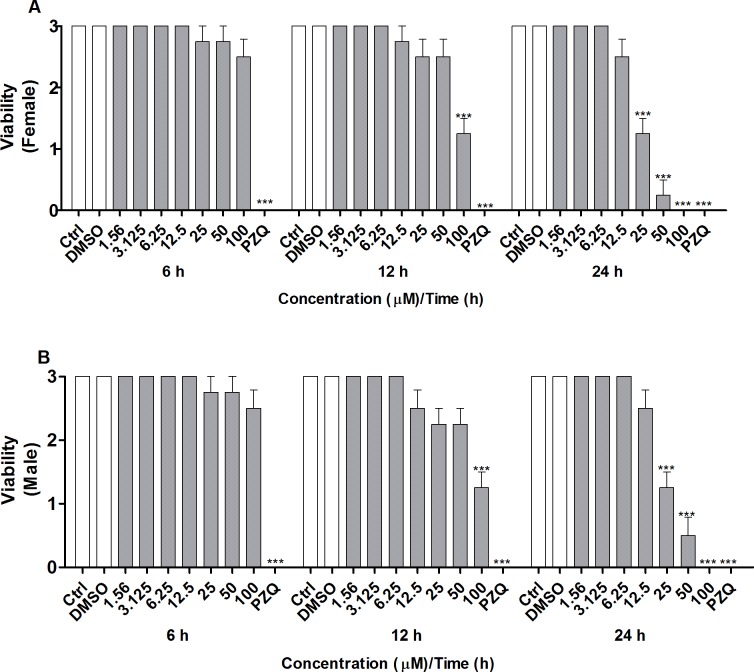
*In vitro* effect of CUR on the viability of adult *S*. *mansoni* worms with emphasis on changes in the motor activity of the worms. Couples of adult worms were incubated with different concentrations of CUR for 6, 12, or 24 h. The viability of separated adult (A) female and (B) male *S*. *mansoni* worms was monitored by using a viability scale of 0–3 (3 = totally vital, normally active, 2 = slowed activity, 1 = minimal activity, 0 = worm death—death was defined as no movement being observed for at least 2 min of examination). In the negative control groups, couples of adult worms were incubated with RMPI 1640 medium or with RPMI 1640 medium with 0.1% DMSO. In the positive control groups, couples of adult worms were incubated with PZQ (1.56 μM). Values are expressed as the mean ± S.E.M of three independent experiments. An asterisk indicates statistically significant differences as compared to the negative control group (RPMI 1640 medium with 0.1% DMSO) (***p < 0.001).

Microscopic observation of couples of adult *S*. *mansoni* worms supported the different viabilities of the worm pairs after exposure to CUR. Viability was assessed on the basis of changes in the motor activity of the worms and of the occurrence of death according to standard procedures for the screening of compounds defined by the WHO-TDR [34]. According to Fig 2A and 2B, CUR significantly decreased the viability of adult female and male worms at the concentrations and incubation times described previously. Mortality was 100% for both female and male worms after exposure to CUR at 100 μM for 24 h. CUR at 25 or 50 μM lowered the motor activity of female and male worms to a minimum or killed them. The LC_50_ values obtained for CUR were 491.0, 90.2, and 32.9 for female worms and 491.0, 90.8, and 43.9 for male worms at 6, 12, and 24 h, respectively. Additionally, CUR at 25 μM separated 75% of the couples of adult worms, whilst CUR at 50 or 100 μM separated all the couples of adult worms after incubation for 24 h (data not shown). The couples of worms in the negative control groups (RPMI 1640 medium alone or in combination with 0.1% DMSO) exhibited normal viability and remained coupled (i.e., the couples of adult worms were not separated), whereas the couples of worms in the positive control group (PZQ at 1.56μM) showed no viability (100% death) and no separation.

### CUR induces alterations in the tegument and organelles of adult *S*. *mansoni* worms

**Fig 3:**
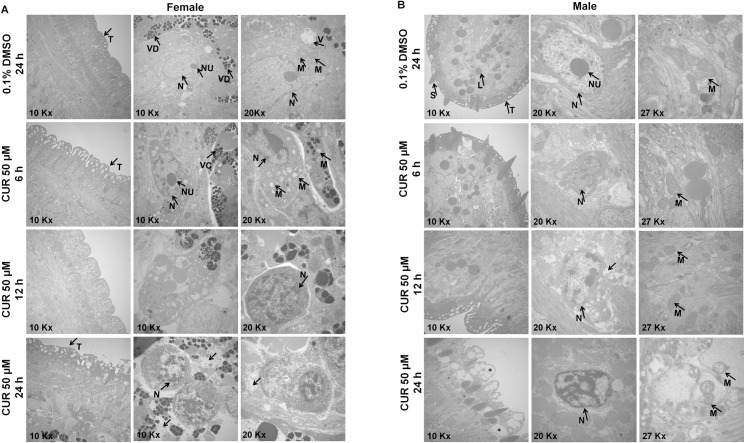
CUR induces alterations in the tegument and organelles of adult *S*. *mansoni* worms. Couples of adult worms were incubated with CUR at 50 μM for 6, 12, or 24 h. After incubation, female and male *S*. *mansoni* worms were separated and processed for Transmission Electron Microscopy (TEM) analysis. In the negative control groups, couples of adult worms were incubated with RPMI 1640 medium with 0.1% DMSO for 24 h. (A) Micrograph of adult female worms and (B) Micrograph of adult male worms. T, tegument; S, spine; L, lipid; N, nucleus, NU, nucleolus; M, Mitochondria; VC, Vitelline cell; VD, Vitelline droplets; Asterisk, vacuolization of the tegument; Arrow, lysis of tissue. A total of 20 adult female and male worms were evaluated at each concentration.

To analyze the ultrastructural alterations, we incubated couples of adult worms with CUR at 50 μM for 6, 12, and 24 h. Adult worms were separated either by action of CUR or manually. Next, we analyzed the worms by TEM. Concerning the adult female *S*. *mansoni* worm in the control group (RPMI 1640 with 0.1% DMSO), the tegument was intact, the muscular layer had preserved fibers throughout the body, most vitelline cells were normal, the cytoplasm was rich in granular endoplasmic reticulum and mitochondria, and vitelline droplets existed inside vitelline balls (Fig 3A). However, some vitelline cells contained small vacuoles, and some nuclear chromatin was undergoing condensation. After incubation for 6 or 12 h with CUR, the mitochondrial membrane swelled, chromatin condensed, and small vacuoles emerged in female *S*. *mansoni* worms, but the tegument remained unaltered. Interestingly, drastic changes occurred in the vitelline cells of female *S*. *mansoni* worms after 24 h of incubation. The interstitial tissue underwent lysis, the mitochondria swelled and degenerated, and chromatin condensed. Additionally, analysis of some parts of the tegument indicated swelling and formation of vacuoles of different sizes (Fig 3A).

The male *S*. *mansoni* worms in the control group (RPMI 1640 with 0.1% DMSO) presented intact tegument and spines with regular morphology. The muscular layer exhibited preserved fibers throughout the body, and the mitochondria and cells had normal morphology (Fig 3B). After 6 h of incubation with CUR, there were no structural alterations in male *S*. *mansoni*; at 12 h of incubation, nuclear chromatin began to condense, but the tegument remained unaltered. However, at 24 h of incubation with CUR, vacuoles of different sizes emerged in the tegument, mitochondria swelled and ruptured, small vacuoles arose, and nuclear chromatin condensed (Fig 3B).

### CUR induces DNA fragmentation and damage in adult *S*. *mansoni* worms

**Fig 4:**
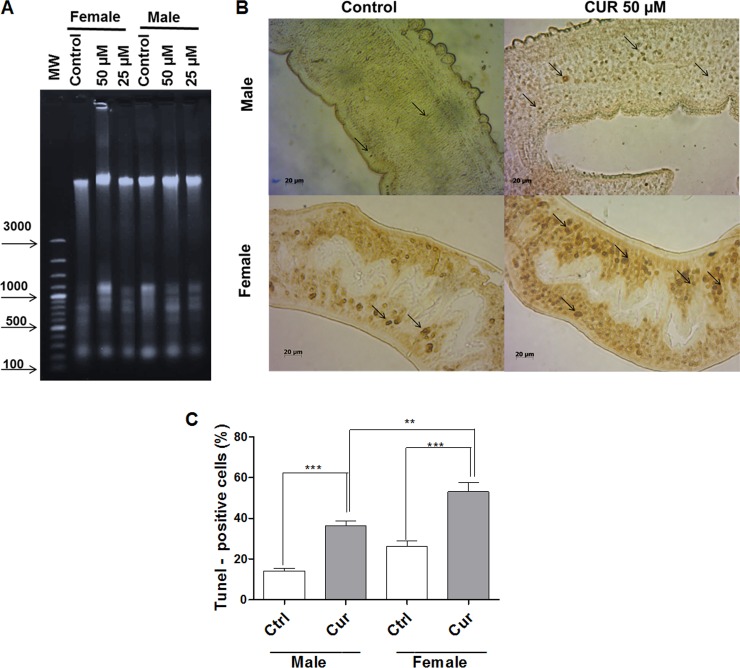
CUR induces DNA fragmentation and damage in adult *S*. *mansoni* worms. Couples of adult worms were incubated with CUR at the indicated concentrations for 24 h. After incubation, female and male *S*. *mansoni* worms were separated and analyzed. In the negative control groups, couples of adult worms were incubated with RPMI 1640 medium with 0.1% DMSO. (A) Genomic DNA of adult female and male worms was extracted as described in material and methods, and 600 ng of the DNA was run in 2% agarose gel containing 1% GelRed (1:500) (MW Molecular weight marker). The experiments were repeated twice, and ten couples of adult worms were evaluated in each experiment. (B) TUNEL-stained light micrographs of adult female and male worm sections (arrows indicate the dark brown-stained apoptotic nuclei). (C) Histograms indicate the percentage of TUNEL-positive cells. For each experiment, at least 100 cells were analyzed. Values are expressed as the mean ± S.E.M of three independent experiments. An asterisk indicates statistically significant differences as compared to the negative control group (RPMI 1640 medium with 0.1% DMSO) or when male and female worms were compared (**p < 0.01, ***p < 0.001).

We incubated couples of adult worms with CUR at 25 or 50 μM for 24 h and then extracted and analyzed the DNA of female and male adult worms (separated either by action of CUR or manually) by electrophoresis on 2% agarose gel. DNA fragmentation slightly increased in the adult female and male worms of the negative control group (worms incubated with RPMI 1640 medium with 0.1% DMSO) (Fig 4A). In contrast, incubation with CUR significantly increased DNA fragmentation in adult female and male worms (Fig 4A). We also evaluated DNA fragmentation by using the Terminal Deoxynucleotidyl Transferase dUTP Nick End Labelling (TUNEL). We incubated couples of adult worms with CUR (50 μM). TUNEL-positive cells (dark brown apoptotic nuclei) increased significantly in adult female and male worms as compared to the negative control group (Fig 4B and 4C). Additionally, TUNEL-positive cells increased more significantly in adult female worms as compared to adult male worms.

We also evaluated DNA damage by the comet assay. The comet assay is an alkaline single-cell gel electrophoresis that is used to measure breaks in DNA strands in cells that are embedded in agarose and submitted to lysis to remove membranes and soluble cell constituents. Lesions can be observed because damaged DNA migrates faster than undamaged DNA. In other words, in cells with damaged DNA, the DNA migrates from the nucleus toward the anode, which resembles the shape of a comet [36]. The frequency of DNA damage increased in female and male cells incubated with CUR at 25 or 50 μM at 24 h as compared to the negative control group (Table 1).

**Table 1:** Frequency of DNA damage in the cells of adult *S*. *mansoni* worms incubated with CUR.

	% Comet class in the cells of female and male adult worms ^*a*^										Score	
	0		1		2		3		4			
Samples	Female	Male	Female	Male	Female	Male	Female	Male	Female	Male	Female	Male
Control^*b*^	67.2±5.2	71.5±6.4	20.1±6.2	18.7±4.3	7.4±4.1	5.3±2.9	6.3±3.0	4.5±2.0	0.0±0.0	0.0±0.0	53.8±23.4	42.8±16.1
25 μM	16.3±4.7	23.0±5.2	59.3±8.7	55.1±8.1	14.0±5.9	13.4±4.2	10.4±2.9	8.5±2.6	0.0±0.0	0.0±0.0	118.5±29.2***	107.4±24.3***
50 μM	13.9±5.8	16.3±6.1	58.1±9.5	53.6±8.3	14.1±6.7	17.5±6.9	13.9±5.4	12.6±4.8	0.0±0.0	0.0±0.0	128.0±39.1***	126.4±36.5***

^*a*^ Fifty random cells were visually analyzed on each slide. Each comet class had a value that ranged between 0 and 4: (0) undamaged cells (all the DNA was located in the head) and (4) maximum damage (almost all the DNA was located in the tail). The total score was calculated by multiplying the percentage of damaged nucleoids by the value of the respective comet class (0, 1, 2, 3, or 4). A value of 0 indicates that no damage occurred; a value of 400 corresponds to maximum damage.

^*b*^Couples of adult worms incubated with RPMI 1640 with 0.1% DMSO. Values are expressed as the mean ± S.E.M of three independent experiments. An asterisk indicates statistically significant differences as compared to the negative control group (RPMI 1640 medium with 0.1% DMSO)

***p < 0.001.

### CUR increases the expression of *SmCASP3/7* transcripts and the activity of Caspase 3 in adult *S*. *mansoni* worms, but the activity of Caspase 8 remains unaltered

**Fig 5:**
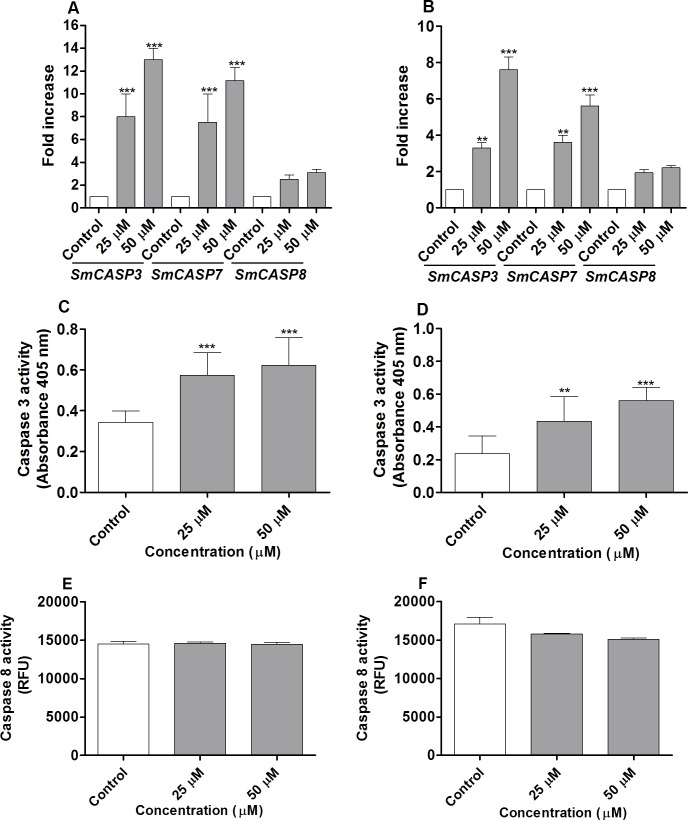
CUR increases the expression of *SmCaspase 3*/*7* and the activity of Caspase 3 in adult *S*. *mansoni* adult worms, but it does not increase the activity of Caspase 8. Couples of adult worms were incubated with CUR at 25 and 50 μM for 24 h. After incubation, female and male *S*. *mansoni* worms were separated and analyzed. In the negative control groups, couples of adult worms were incubated with RPMI 1640 medium with 0.1% DMSO. Relative levels of expression of *SmCASP3*, *SmCASP7*, and *SmCASP8* transcripts in adult (A) female and (B) male worms incubated with CUR. Expression was calculated according to the comparative Ct method (2^-ΔΔCT^ method), and data were normalized relative to an endogenous standard gene (*SmGPDH)*. The activities of Caspase 3 and 8 were measured as described in material and methods. The activity of Caspase 3 referred to adult (C) female and (D) male worms. The activity of caspase 8 referred to adult (E) female and (F) male worms. Values are expressed as the mean ± S.E.M of three independent experiments. An asterisk indicates statistically significant differences as compared to the negative control group (RPMI 1640 medium with 0.1% DMSO) (**p < 0.01, ***p < 0.001).

To test whether CUR induces cell death, we determined the levels of expression of *SmCASP3*, *7*, and *8* transcripts by quantitative RT-PCR in adult worms separated either by action of CUR or manually. *SmCASP3/7* increased significantly in female and male worms incubated with CUR at 25 or 50 μM for 24 h as compared to the negative control group (worms incubated with RPMI 1640 medium plus 0.1% DMSO). At higher concentration of CUR (50 μM), the *SmCASP3* and *SmCASP7* transcripts were upregulated by about 13- and 11.1-fold in adult female worms, respectively (Fig 5A). In adult male worms incubated with CUR, the *SmCASP3* and *SmCASP7* transcripts were upregulated by about 7.6- and 5.7-fold, respectively (Fig 5B). On the other hand, analysis of the transcription levels showed *SmCASP8* increased only slightly in both female and male worms incubated with CUR as compared to the transcription levels of *SmCASP3* and *SmCASP7* (Fig 5A and 5B). These results resembled the data reported by Morais et al. [27], who demonstrated about twofold upregulation for *SmCASP8*.

We also analyzed how the activities of Caspase 3 and 8 changed in adult worms incubated with CUR at 25 or 50 μM. The activity of Caspase 3 increased significantly in both adult female and male *S*. *mansoni* worms after incubation with CUR for 24 h as compared to the negative control group. At higher concentration of CUR (50 μM), the activity of Caspase 3 increased by more than 80% in adult female and male worms (Fig 5C and 5D). On the other hand, the activity of Caspase 8 remained unaltered in both female and male *S*. *mansoni* worms (Fig 5E and 5F).

### CUR induces formation of the superoxide anion and increases the activity of SOD activity in adult *S*. *mansoni* worms

**Fig 6:**
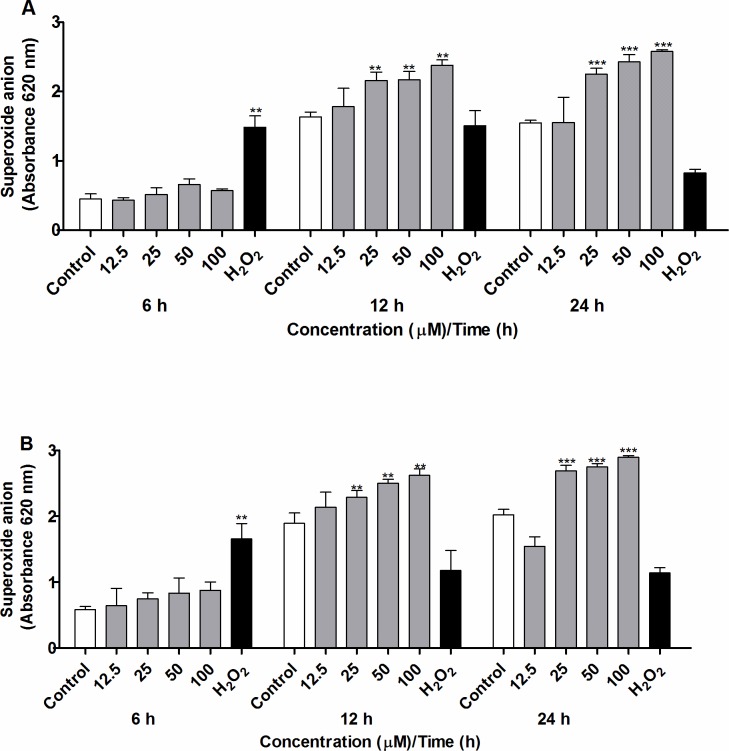
CUR induces formation of the superoxide anion in adult *S*. *mansoni* worms. Couples of adult worms were incubated with CUR at 12.5 to 100 μM for 6, 12, or 24 h. After incubation, (A) female and (B) male *S*. *mansoni* worms were separated, and the level of superoxide anion was measured by the NBT assay at 620 nm. Couples of adult worms incubated with RPMI 1640 with 0.1% DMSO were used as negative control, and couples of adult worms incubated with RPMI 1640 medium with 100 μM hydrogen peroxide were used as positive control. The results represent the mean ± SEM of three independent experiments. An asterisk indicates statistically significant differences as compared to the negative control group (RPMI 1640 medium with 0.1% DMSO) (**p < 0.01, ***p< 0.001).

We incubated pairs of adult worms with CUR at 12.5–100 μM for 6, 12, or 24 h and evaluated the level of superoxide anion in adult female and male worms separated either by action of CUR or manually. We employed the colorimetric Nitroblue Tetrazolium (NBT) assay during this analysis [43]. The level of superoxide anion increased significantly in adult female and male *S*. *mansoni* worms incubated with CUR at 25 to 100 μM after 12 or 24 h of incubation as compared to the negative control group (worms incubated with RPMI 1640 medium with 0.1% DMSO). For incubations with CUR at 25, 50, or 100 μM for 24 h, the level of superoxide anion increased by more than 44%, 56%, and 66% in adult female worms, respectively, and 34%, 37%, and 44% in adult male worms, respectively. On the other hand, 6 h of incubation with CUR did not alter the level of the superoxide anion as compared to the negative control group (Fig 6A and 6B). Both male and female worms in the positive control group had significantly increased levels of the superoxide anion at 6 h.

Inside the cells, the superoxide anion (O_2_^-^) is converted to hydrogen peroxide (H_2_O_2_) in a reaction catalyzed by the enzyme SOD. Here, we determined the activity of SOD in female and male adult worms incubated with CUR at 25 or 50 μM for 6, 12, or 24 h. As shown in Table 2, 6 and 12 h of incubation with CUR did not change the activity of SOD significantly in adult female and male worms as compared to the negative control group (worms incubated with RPMI 1640 medium with 0.1% DMSO). In contrast, the activity of SOD increased significantly in adult female and male worms 74% and 58%, respectively at 24 h of incubation (Table 2). The activity of SOD was also significantly higher in the positive control at 6, 12, and 24 h in both male and female worms (data not shown).

**Table 2:** Effect of CUR on the activity of different antioxidant enzymes in adult *S*. *mansoni* worms.

Antioxidant enzymes	Enzymatic activities at the following hours of incubation with CUR or control^*a*^								
	6 h			12 h			24 h		
	Control^*b*^	25 μM	50 μM	Control^*b*^	25 μM	50 μM	Control^*b*^	25 μM	50 μM
Female									
SOD	2.8±0.9	2.9±0.7	3.0±0.9	3.1±0.9	3.6±0.6	3.8±0.1	3.1±0.8	4.7±0.3***	5.4±0.4***
		(+3.5%)	(+7.1%)		(+16.1%)	(+22.5%)		(+51.6%)	(+74.1)
GST	17.3±1.9	17.2±2.1	16.9±3.2	19.0±1.7	18.3±1.5	17.3±1.2	20.2±2.5	14.6±3.2***	13.7±3.1***
		(-0.6%)	(-2.3%)		(-3.8%)	(-8.9%)		(-27.7%)	(-32.1%)
GR	11.1±1.2	11.5±0.6	9.6±1.6	11.8±2.3	10.8±1.7	9.0±1.1	11.4±0.9	7.7±1.3***	5.1±1.8***
		(+3.6%)	(-13.5%)		(-8.4%)	(-16.6%)		(-32.4%)	(-55.2%)
GPX	45.9±4.5	43.1±2.6	42.9±3.1	46.7±2.2	44.0±2.9	42.7±5.0	49.9±3.3	30.2±3.6***	29.8±4.6***
		(-6.1%)	(-6.5%)		(-5.7%)	(-8.5%)		(-39.4%)	(-40.2%)
Male									
SOD	2.5±0.2	2.6±0.4	2.8±0.7	2.7±0.5	3.0±0.8	3.1±0.7	3.4±0.4	4.9±0.5***	5.4±0.6***
		(+4.0%)	(+12%)		(+11.1%)	(+14.8%)		(+44.1%)	(+58.8%)
GST	19.6±1.7	18.9±2.1	18.1±1.6	19.3±2.9	18.3±1.3	17.4±2.6	20.4±3.2	15.8±4.3***	14.8±3.***
		(-3.6%)	(-7.6%)		(-5.9%)	(-9.8%)		(-22.5%)	(-27.4%)
GR	10.5±0.6	9.6±1.2	9.1±0.8	11.7±2.1	10.8±0.6	10.5±1.4	12.5±1.1	7.2±0.7***	4.6±1.0***
		(-8.5%)	(-13.3%)		(-14.9%)	(-17.3%)		(-42.4.%)	(-63.2%)
GPX	48.7±2.1	48.1±3.7	45.5±1.8	49.5±3.1	41.2±1.8	40.4±2.4	50.3±2.9	34.5±1.6***	30.7±2.7***
		(-1.2%)	(-6.5%)		(-16.7%)	(-18.3%)		(-31.4%)	(-38.9%)

^*a*^Couples of adult worms were incubated with CUR at 25 or 50 μM for 6, 12, or 24 h. The activities of the antioxidant enzymes were measured as described the materials and methods section. Values in parentheses indicate the percentage of inhibition/activation as compared to the negative control group.

^*b*^ Pairs of adult worms incubated with RPMI 1640 with 0.1% DMSO. Activities are indicated as U/mg, and the values are expressed as the mean ± S.E.M of three independent experiments. An asterisk indicates statistically significant differences as compared to the negative control group (RPMI 1640 medium with 0.1% DMSO)

***p < 0.001.

### CUR alters various oxidative stress parameters in adult *S*. *mansoni* worms

To investigate other oxidative stress parameters, we incubated pairs of adult *S*. *mansoni* worms with CUR at 25 or 50 μM for 6, 12, or 24 h and evaluated the activities of GST, GR, and GPX and the levels of protein carbonyl in both adult female and male worms separated either by action of CUR or manually. Table 2 shows that the activities of GST, GR, and GPX decreased significantly in adult female and male worms after 24 h of incubation with CUR at 25 or 50 μM. CUR at 50 μM inhibited the activity of GST in adult female and male worms by more than 32% and 27%, respectively. CUR at 50 μM inhibited the activity of GR in adult female and male worms by more than 63% and 55%, respectively. Finally, CUR at 50 μM inhibited the activity of GPX in adult female and male worms by more than 40% and 38%, respectively. At 6 and 12 h of incubation, the activities of GST, GR, and GPX did not decrease significantly as compared to the negative control group. In the positive control group (100 μM hydrogen peroxide), the activity of GST increased significantly in female and male worms at 6, 12, and 24 h (data not shown). On the other hand, the activities of GR and GPX decreased significantly in both adult female and male worms (data not shown) for all the incubation periods.

The levels of protein carbonyl increased significantly in both adult female and male worms after incubation with CUR at 25 or 50 μM for 24 h as compared to the negative control group. At 6 and 12 h, the levels of protein carbonyl did not change significantly (Table 3). Both male and female worms in the positive control group had significantly increased levels of protein carbonyl at 12 and 24 h of incubation (data not shown).

**Table 3:** Effects of CUR on the content of protein carbonyl in adult *S*. *mansoni* worms.

Samples	Content of protein carbonyl at the following hours of incubation with CUR or control^*a*^					
	6 h		12h		24 h	
	Female	Male	Female	Male	Female	Male
Control ^*b*^	23.3±4.7	26.1±2.8	23.9±2.8	27.0±3.1	25.6±2.8	28.9±3.2
25 μM	23.9±1.6	27.5±3.6	27.3±2.0	30.5±4.1	32.7±2.1***	35.4±4.5***
	(+2.5%)	(+5.3%)	(+14.2%)	(+12.9%)	(+27.7%)	(+22.4%)
50 μM	25.8±3.7	28.1±2.1	28.3±3.2	31.2±2.7	36.9±3.4***	37.8±3.2***
	(+7.9%)	(+7.6%)	(+18.4%)	(+15.5%)	(+44.1%)	(30.7%)

^*a*^ Couples of adult worms were incubated with CUR at 25 or 50 μM for 6, 12, or 24 h. The content of protein carbonyl was measured as described in the materials and methods section. Values in parentheses indicate the increase in percentage as compared to the control.

^*b*^ Couples of adult worms incubated with RPMI 1640 with 0.1% DMSO. The content of protein carbonyl is expressed as mmol/mg protein, and the values are expressed as the mean ± S.E.M of three independent experiments. An asterisk indicates statistically significant differences as compared to the negative control group (RPMI 1640 medium with 0.1% DMSO)

***p < 0.001.

## Discussion

The only schistosomicidal drug that is commercially available is Praziquantel, which has been used in monotherapy for several decades. Therefore, widespread emergence of drug resistance is probable and makes the development of new therapeutic approaches against schistosomiasis mandatory. In the past years, numerous natural products and synthetic drugs have been evaluated as potential schistosomicidal agents [18,19], but few studies have shown the effects of these drugs on the induction of apoptosis in this parasite. Curcumin (CUR), a secondary metabolite of turmeric derived from *C*. *longa* L., displays many biological activities [20,48]. Previous studies have suggested that CUR and CUR analogs can generate reactive oxygen species (ROS) in several cancer cell lines and trigger the apoptotic death of these cells [49–51]. Additionally, CUR exhibits potent anti-filarial effects on a filarial parasite because it induces apoptosis by a mitochondrial pathway [8,31]. Here, we demonstrated that CUR affected oxidative stress and induction of apoptosis in adult *S*. *mansoni* worms.

Previous investigations by our group have demonstrated that CUR at 50 or 100 μM after 24 or 120 h of incubation kills all the couples of adult worms [21]. Based on these results, here we report additional data on the viability of adult worms after conducting the MTT assay and microscopic analyses of pairs of adult worms incubated with CUR for 6, 12, or 24 h. The results suggested potential *in vitro* activity of CUR against female and male *S*. *mansoni* worms at 24 h. However, CUR was more lethal to female worms, LC_50_ values were 32.9 μM as compared to 43.9 μM in the case of adult male worms.

Ultrastructural analysis showed altered tegument in adult female and male worms after incubation with CUR. The tegument is an essential structure for the survival and maintenance of *Schistosoma* worms because it plays a vital role in evading the host’s immune system, acquiring nutrients, excreting catabolic products, and targeting drug absorption, among other physiological processes [52].

Morphologically, cells killed by apoptosis may exhibit morphological changes such as swelled and ruptured mitochondria, condensed chromatin, and fragmented DNA [53,54]. The ultrastructural analysis also demonstrated alterations such as swelling and degeneration of the mitochondrial membrane, condensation of chromatin, and formation of vacuoles in adult female and male worms. In adult female worms, the alterations occurred mainly in the vitellarium, which is a proliferative tissue that occupies the posterior two thirds of the female and produces cells that surround the ovum and provide the precursor proteins that form the eggshell and the nutrients that aid development of the embryo [55]. Additionally, at 24 h, there were some alterations in vitelline cells in adult female worms belonging to the negative control group, as already described by Galanti et al. (2012) [56]. Because the production of eggs is crucial to both the transmission and the pathogenesis of schistosomiasis, drug-induced alterations in the reproductive development of schistosomes could constitute a new method to prevent or treat the disease [55, 56]. Interestingly, our group has previously shown that CUR reduces the production of eggs by more than 50% as compared to the negative control group under *in vitro* conditions [21]. Additionally, Mohapatra et al. (2011) [8] have demonstrated that CUR is the most effective pharmacological agent to induce apoptosis in embryonic stages of the nematode *Setaria digitata*, suggesting that blockage of embryogenesis through therapeutic induction of apoptosis during the embryonic stages of parasites is a promising strategy to develop effective anti-parasitic measures against extracellular parasites.

Considering the alterations observed by TEM, we evaluated different apoptotic parameters in adult worms incubated with CUR. DNA fragmentation is one of the main parameters used to identify cell apoptosis—activated caspases cleave the DNA of the cell, to produce DNA fragments [54]. Evaluation of DNA fragmentation by electrophoresis on agarose gel evidenced that CUR fragmented the DNA of adult female and male worms. In addition, TUNEL staining, a method that detects DNA fragmentation *in situ* (a hallmark of apoptosis), demonstrated that TUNEL-positive cells increased in adult female and male worms as compared to the negative control group. In addition, the number of TUNEL-positive cells was higher in adult female worms as compared to adult male worms. Moreover, the comet assay revealed that CUR damaged DNA in adult female and male worms. Studies have suggested that CUR can fragment and damage DNA [31,57–58]. For example CUR induces *in situ* DNA fragmentation in both embryos and adult females of the parasite *S*. *servi* [31]. Other studies have shown that CUR induces apoptosis in human hepatocellular carcinoma J5 cells [58].

Caspases are pro-apoptotic cysteinyl aspartate protease proteins that are essential to the typical nuclear features of apoptosis in mammalian cells [59]. Four caspases have been described in schistosomes: *SmCASPD*, which shows homology with *CASP2* and *CASP9* (human initiator caspases); *SmCASP3* and *SmCASP7*, which are homologues of the human effector caspases *CASP*3 and *CASP*7, respectively; and *SmCASPC*, which is a homologue of human *CASP8* [16–17, 60–61]. In the present study, we evaluated how CUR affected the expression of transcripts of *SmCASP3*, *7*, and *8* as well as the activities of Caspase 3 and 8 in adult female and male worms. *SmCASP3/7* transcripts and the activity of caspase 3 were upregulated in female and male adult worms after incubation with CUR at 25 or 50 μM. Moreover, the expression of *SmCASP3/7* and the activity of Caspase 3 were higher in adult female worms as compared to adult male worms. The extrinsic pathway that activates caspase starts through binding to death receptors on the cell membrane, to recruit the cytosolic adapter protein FADD and Caspase 8 and to form the death-inducing signaling complex (DISC) that cleaves and activates caspase 3 [9]. Here, the activity of caspase 8 did not increase, suggesting that the activity of caspase 3 could be associated with the induction of apoptosis by the intrinsic pathway. Works by other authors have also suggested that CUR can upregulate the expression of *CASP*3, and that this caspase plays a crucial role in CUR-induced apoptosis in filarial parasites by the intrinsic pathway [8,31].

Mitochondria are the major source and target of intracellular reactive oxygen species (ROS) [8,61–63]. During apoptosis, induced by a variety of stimuli, the permeability of the mitochondrial membrane increases and triggers the release of pro-apoptotic factors including cytochrome-c and AIF (Apoptosis-inducing Factor) into the cytosol. The cytosolic cytochrome-c then interacts with APAF-1 and forms an apoptosome. Activation of the apoptosome can ultimately activate effector caspase and apoptosis [63–65]. A wide range of chemicals or natural compounds can stimulate the formation of ROS and trigger apoptosis [66–67]. High concentrations of CUR (at 25 and 50 μM) promote the formation of ROS in different cell lines, whereas low CUR (at 10 μM) usually diminishes the formation of ROS [28, 68–71]. The results of the present study were consistent with data reported by other authors [8, 31, 49–51] and showedhat CUR and some of its derivatives induced production of ROS.

In schistosomes and other helminthic parasites, diverse antioxidant systems that depend on antioxidant enzymes, or not, regulate the concentrations of ROS inside the cell. Adult *S*. *mansoni* worms have a long life span and constantly face stress conditions within the host, so they have evolved a series of antioxidant enzymes to evade the host’s hostile environment [72]. Among these enzymes are SOD, GST, GR, and GPX, which play an essential part in balancing the production and decomposition of ROS and in protecting the parasite from damage as a result of enhanced production of ROS like superoxide radical anion and hydroxyl radicals [73,74]. The induction of oxidative stress due to abatement of the antioxidant system or to increased production of ROS in adult *S*. *mansoni* has been considered an attractive approach to new treatment strategies [74–76].

Enzymatic antioxidants such as GST and GR act as the first line of a defense mechanism and maintain the cellular redox system by scavenging the free radicals. In schistosomes, GSTs can induce passive detoxification of antischistosomal drugs and haematin [77,78]. Also, GST enzymes might become increasingly exposed during drug-induced tegumental and subtegumental damage [79]. In *S*. *mansoni* worms, GSTs are mainly located in the subtegumental parenchyma [78,79]. In the present study, the activity of SOD increased and the activities of GST, GR, and GPX decreased in both adult female and male worms incubated with CUR. However, the effects on the activities of these enzymes were more pronounced in adult female worms as compared to adult male worms. A previous work that used microarray analysis showed that the *SmSOD 1* transcript increased in *S*. *mansoni* after incubation with CUR [27]. In addition, studies have shown that CUR can increase the activities of SOD and GST in the several tumor cells and filarial parasite [31, 80]. Oxidative stress is harmful to cells, it causes oxidative modification of cellular macromolecules or alters the normal function of proteins, promoting cell death. Our study evidenced increased content of protein carbonyl content in adult female and male worms incubated with CUR.

In summary, the results of the present work suggest that CUR generates oxidative stress followed by an apoptotic-like event in adult female and male *S*. *mansoni* worms, which ultimately leads to parasite death. Induction of apoptotic death is a therapeutic approach that needs to be further explored during the development of new drugs with broad spectrum and anthelminthic activity.
